# Full-Body Kinematics and Vertical Ground Reaction Forces in Elite Ten-Pin Bowling: A Field Study

**DOI:** 10.3390/s23198284

**Published:** 2023-10-07

**Authors:** Bo Eitel Seiferheld, Jeppe Frost, Thorstein Brynildsen Østergaard, Mathias Sønder Krog, Kent Kongsøre Klitgaard, Mark de Zee

**Affiliations:** 1 Biomechanics, Department of Materials and Production, Aalborg University, Fibigerstræde 16, 9220 Aalborg East, Denmark; bes@mp.aau.dk; 2 ExerciseTech, Department of Health Science and Technology, Aalborg University, Selma Lagerløfs Vej 249, 9260 Gistrup, Denmarkklit@hst.aau.dk (K.K.K.)

**Keywords:** ten-pin bowling, biomechanics, kinematics, vertical ground reaction forces, performance, SPM analysis, wearable sensors

## Abstract

The purpose was to investigate full-body kinematics and vertical ground reaction forces in the lower extremities of the delivery and to determine delivery changes over time after many deliveries in ten-pin bowling. Six male elite ten-pin bowlers completed six bouts of twelve bowling deliveries, all strike attempts, while measuring full-body kinematics and vertical ground reaction forces. Full-body joint angles, peak vertical ground reaction forces in the feet, vertical breaking impulse, centre of mass velocity, bowling score, and ball release velocity (BR_vel_) were measured. Results revealed that the BR_vel_ was significantly decreased over bouts (*p* < 0.001). Additionally, increased flexion of the dominant wrist (*p* < 0.001) and elbow (*p* = 0.004) prior to ball release (BR) and increased pronation of the dominant wrist during BR (*p* = 0.034) were observed at later bouts. It was concluded that these kinematic changes in the dominant wrist and elbow prior to and during BR were performed to compensate for the change in traction between ball and lane during a bowling match. This, in turn, caused a decrease in BR_vel_. A conservation of energy perspective was discussed to highlight training applications and possibilities to enhance elite athletes’ bowling performance.

## 1. Introduction

In ten-pin bowling, 10 pins are placed in a tetractys formation at the end of an 18.3 m long wooden lane. The objective for the bowler is to knock down as many pins as possible, rolling a bowling ball down the wooden lane. Two attempts are given to each player before resetting the pins. Knocking down all pins in the first attempt is called a strike, and results in the best score. A bowling delivery consists of five steps, starting on the left foot for right-handed players. Firstly, the bowler initiates a run-up phase and in-between the third last and second last heel touch, the bowling ball reaches the top of the backswing (TBS), the position where the bowling ball has the highest potential energy. From the TBS, the bowling ball follows a pendulum trajectory transforming the potential energy to kinetic energy. As the pendulum motion occurs, during the second last heel touch, the bowler pushes with the dominant foot into the final heel touch known as the front foot slide (FFS). During the FFS, the bowler slides with the non-dominant foot, and breaks before a foul line and releases the bowling ball [1].

Psychologically, skilled bowlers demonstrate greater mental toughness, and more confidence in performing the techniques and handling of equipment, than less skilled bowlers [2]. Whereas physiological components such as grip strength, 10 repetition max leg press, aerobic power index [3,4], and age-related performance decreases [5] are less critical factors in ten-pin bowling, compared to other sports disciplines like running and swimming [6,7]. Thus, it is conceivable that ten-pin bowling is a sports discipline that requires a high level of technique, and the field of biomechanics may be a way to provide coaches and athletes with valuable information to improve performance.

An attempt to profile elite ten-pin bowlers in delivery techniques was conducted by Chu et al. [1]. Here, kinematic differences were observed in the sagittal plane between male and female bowlers. Males reached a higher TBS than females, whereas females came closer to the toe foul line [1]. Newer observations highlighted the importance of generating a high bowling ball release velocity (BR_vel_), as this was significantly correlated with the average bowling score achieved during the bowlers last three tournaments (r = 0.59) [8]. Additionally, BR_vel_ was significantly correlated with the peak foot velocity (r = 0.52), peak acceleration (r = 0.39), and peak deceleration (r = −0.48) of the FFS [8]. Moreover, temporal characteristics in terms of the time from the start of the FFS until the ball release (BR) was found to be negatively correlated with BR_vel_ (r = −0.414) [9]. In cricket, similarities have been observed to bowling. Here, the velocity of the players’ centre of mass (COM_vel_) and the horizontal breaking impulses seem to be associated with the ability to generate higher BR_vel_ [10,11,12]. Current knowledge within ten-pin bowling suggests that the initial velocity, the execution time of the delivery phase, and the skill to generate a high BR_vel_ is desirable.

Published work about the biomechanics of ten-pin bowling is relatively scarce. Therefore, this two-pronged study aimed to investigate full-body kinematics and vertical ground reaction forces in the lower extremities of the delivery and to determine delivery changes over time after many deliveries in ten-pin bowling. An explorative approach was chosen for the full-body kinematics analysis. However, it was still hypothesised that (I) the BR_vel_ is positively correlated with the average bowling score; (II) vertical breaking impulse (BR_imp_) is negatively correlated with BR_vel_; (III) COM_vel_ of the bowler is positively correlated with BR_vel_.

## 2. Materials and Methods

### 2.1. Participants

Six members of the male Danish national bowling team participated in the study (1.88 ± 0.05 m, 92.7 ± 16 kg, 26.1 ± 5 years, 20.3 ± 7.4 years of bowling experience), all right-handed who identified themselves as being Tweener style bowlers. All participants had international experience and a season bowling average minimum of 200 pins. None of the participants had any history of injuries affecting their bowling performance one month prior to data collection. All participants received written and oral information about the procedures and the overall aim of the study and subsequently provided written informed consent. The experimental data were collected following the standard ethical guidelines of The Scientific Ethical Committee for the Region of Northern Jutland.

### 2.2. Experimental Protocol

The bowling performance of the participants was investigated in six consecutive bouts of 12 bowling trials, resulting in a total of 72 trials for each participant. The choice of 12 trials per bout was based on a perfect game of only strikes and six bouts were to simulate the actual length of a match. The bouts were separated by a five-minute rest period to avoid the risk of fatigue during recordings and participants aimed for a strike at every trial, resetting pins afterwards. All participants used their normal personal ball used in competitions, which was a 7.5 kg bowling ball, and were not allowed to change it during the recordings. Before initiating the first bout, participants carried out a 10 min warm-up and acclimation period on an adjacent bowling lane to become familiar with the equipment used for data recording.

### 2.3. Data Recording

**Figure 1:**
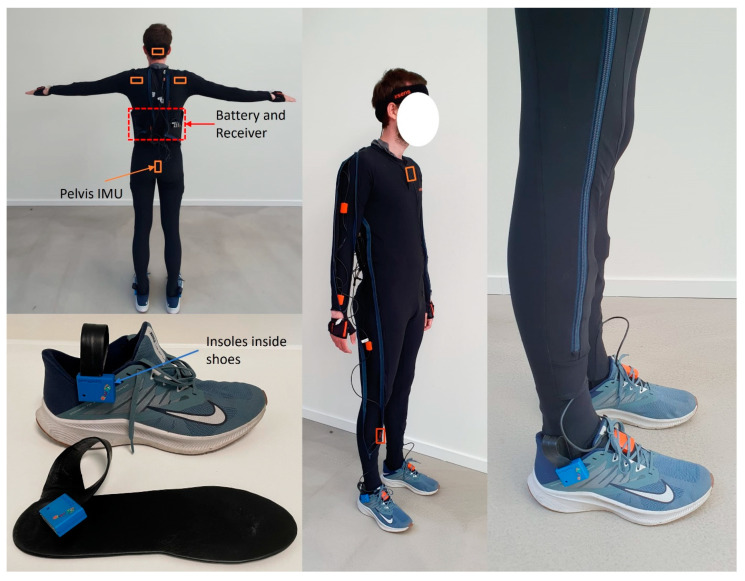
Instrumentation of the 17 IMU sensors (orange boxes), battery, receiver, and shoe insoles. Please note that IMUs were placed on the interior of the suit and symmetrical between the limbs but the suit is unzipped for visualisation purposes.

At every trial, kinematic data were captured by a validated system based on inertial measurement units (IMU), the Xsens Link (Xsens Technologies BV, Enschede, The Netherlands), sampling at 240 Hz. IMUs were placed on seventeen body segments: head, sternum, shoulders, upper arms, lower arm, hands, pelvis, thighs, shanks, and each foot (Figure 1) and anthropometric data were measured. The Xsens system was calibrated using the N-pose feature, and proprietary sensor fusion algorithms were then used to provide joint angles [13]. The Loadsol^®®^ insole force sensor system (Novel GmbH, Munich, Germany) sampled vertical ground reaction forces (GRF) at 100 Hz during bowling deliveries. A thin, single capacitor sensor insole was inserted into the participants’ shoes and fitted to their shoe size (Figure 1). The data acquisition box, which communicates and transfers data wirelessly via Bluetooth to a smartphone, was clipped to the collar of the shoes to decrease movement interference. The system was calibrated by loading and unloading the insole in a single-leg stance. Calibration was performed after the warm-up to ensure that potential heating and fluid produced within the shoes would not affect the sensitivity of the measurements. The Loadsol insole force sensors were validated during controlled experiments using force plates as golden standard and the insoles were used to measure GRFs during different movement [14,15]. Both systems could detect steps independently, and the onset and offset of the steps were used to synchronize the data streams. A laser-based tracking system installed in bowling alleys, Specto (Kegel version 1.0. Lake Wales, FL, USA), was used to measure the bowling score (B_score_) as pins (0–10) and BR_vel_. Specto was validated before data recording. Briefly, BR_vel_ was measured with Specto and TC-System photogates (Brower Timing System, Draper, UT, USA), simultaneously while 20 bowling ball trials were performed at three different speeds: low, medium, and high velocity. An unpaired t-test found no significant difference between these means [t (118) = 0.034, *p* = 0.973] and the correlation between the systems was almost perfect (r = 0.99, R2 = 0.99).

### 2.4. Data Analysis

#### 2.4.1. Bowling Parameters

**Figure 2:**
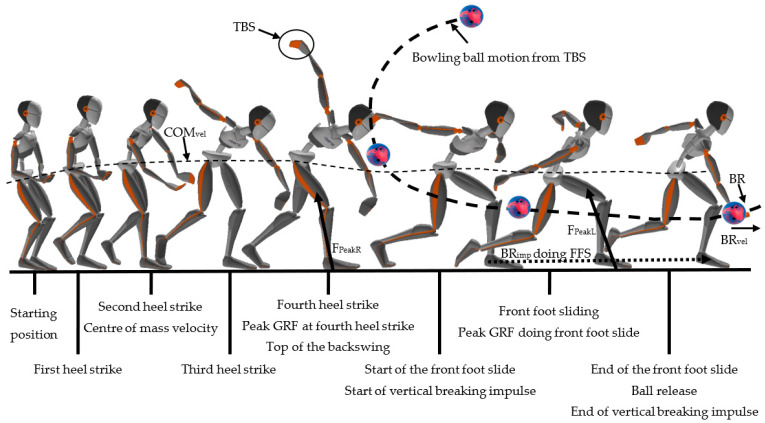
A comprehensive representation of a typical bowling ball delivery with the right hand, demonstrating the sequence of events and important parameters throughout the process. Showing: centre of mass velocity (COM_vel_), top of the backswing (TBS), peak vertical ground reaction force for the last heel strike before starting the front foot slide (F_PeakR_) and performing the front foot slide (F_PeakL_), vertical breaking impulse calculated from the start of the front foot slide until ball release, front foot slide (FFS), ball release (BR), and ball release velocity (BR_vel_).

For every bowling trial, peak vertical GRF in the left (F_peakL_) and right (F_peakR_) feet, vertical BR_imp_, COM_vel_, B_score,_ and BR_vel_ were quantified as bowling parameters (Figure 2). To calculate BR_imp_, the FFS duration was determined to start at initial foot contact of the sliding leg (left), as it exceeded 20 N and end at BR. BR was determined as the resultant peak acceleration of the dominant hand during the ball delivery. The assumption for determining BR was based on Newton’s second law; during the delivery, the bowling ball leaves the hand, which causes a sudden increase in acceleration of the hand due to a sudden mass reduction. Peak forces were normalised to percentage body weight (%BW) and impulse to %BW per second (%BW s) to enable comparison independent of body weight [16].

#### 2.4.2. Joint Angles

To ensure that differences in anthropometrics and possible differences in execution times would not influence the results, shoulder, elbow, wrist, hip, knee, and ankle joint angles were extracted with respect to their movements (e.g., flexion/extension) and data were time-normalised to 0–100% of a bowling delivery. The data analysis was performed using MATLAB version 2018b (MathWorks Inc., Natick, MA, USA). 

### 2.5. Statistical Analysis

One-way repeated measures ANOVA, with least significant differences correction, was performed to analyse differences between the dependent variables F_peakL_, F_peakR_, BR_imp_, COM_vel_, B_score,_ and BR_vel_. Pearson product-moment correlation coefficient was calculated, and interpretation of the correlation coefficient size was rated as follows: <0.25 weak, 0.25 to 0.50 moderate, 0.51 to 0.75 fair, and >0.76 high [17]. Linearity was tested for significance with an F-test. Statistical tests were conducted using SPSS version 25 (SPSS Inc., Chicago, IL, USA). Changes in movement patterns were investigated with open source one-dimensional statistical parametric mapping (SPM1D) and one-way repeated measures ANOVA, which assesses continuous data over time [18,19], coded (v.0.4, www.spm1d.org, accessed on 17 October 2020) in MATLAB. Data are presented as mean ± standard deviation (SD) unless stated otherwise. The significance level was set at *p* < 0.05.

## 3. Results

### 3.1. Bowling Parameters

Inspecting the bowling parameters revealed a significant main effect of bouts for BR_vel_ (*p* < 0.001) (Table 1). Post hoc analysis revealed that bout 6 was significantly lower than bout 1 (*p* = 0.012), bout 2 (*p* = 0.008) and bout 3 (*p* = 0.039). In addition, bout 5 was significantly lower than bout 1 (*p* = 0.028). No main effects were observed between any of the other measured bowling parameters. Bowlers reached an average height of 1.69 ± 0.22 m at TBS and spent on average 640 ± 95 ms until BR. Bowling deliveries were performed consistently within bouts as presented with a relatively low intra-individual variation in the bowling parameters for bout 1 (Table 2).

### 3.2. Correlation Matrix

The correlation analysis was based on the mean values of each participant, giving a total of six data points. Each point was comprised of 72 bowling trials for one participant and results revealed no significant correlations (Table 3). A high positive correlation between F_peakL_ and BR_imp_ (r = 0.80, *p* = 0.057), and a high negative correlation between F_peakR_ and BR_vel_ (r = −0.80, *p* = 0.057) was observed, both borderline significant. The relationship between BR_vel_ and B_score_ was positive (r = 0.32, *p* = 0.54). BR_imp_ and BR_vel_ were negatively related (−0.21, *p* = 0.68); also, COM_vel_ and BR_vel_ were negatively correlated (−0.45, *p* = 0.37).

### 3.3. Joint Kinematics

**Figure 3:**
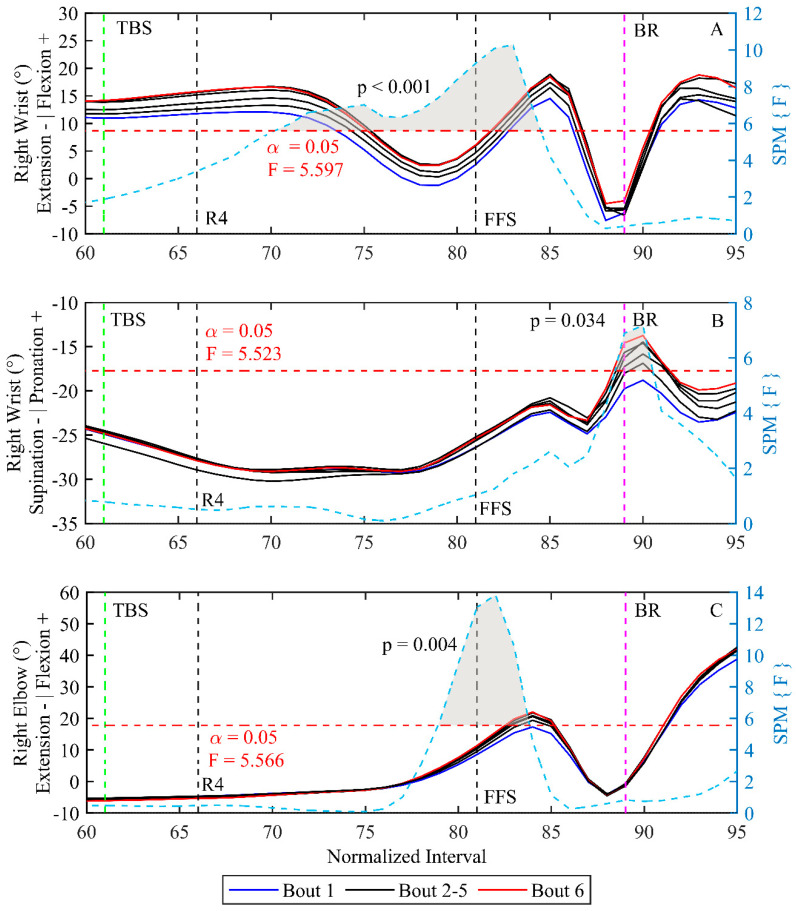
Shows mean angles for all participants throughout bouts. Bout one is blue, with bouts in-between being black and bout six is red. Black vertical lines are mean heel contact of right (R4) as the fourth step and FFS as the left and last step of the bowling delivery. Green and pink lines represent TBS and BR, respectively. SPM1D analysis is inserted onto the figure as a dashed blue line; the critical threshold is presented as a horizontal red dashed line, with an alpha value of 0.05 and a corresponding F-value. Clusters of significant differences are marked with grey and a *p*-value. Be aware that the statistics show within subjects, whereas the bouts are an average of all subjects; further SD values are omitted for clarity. (**A**) Flexion/extension of the right wrist. (**B**) Pronation/supination of the right wrist. (**C**) Flexion/extension of the right elbow.

**Figure 4:**
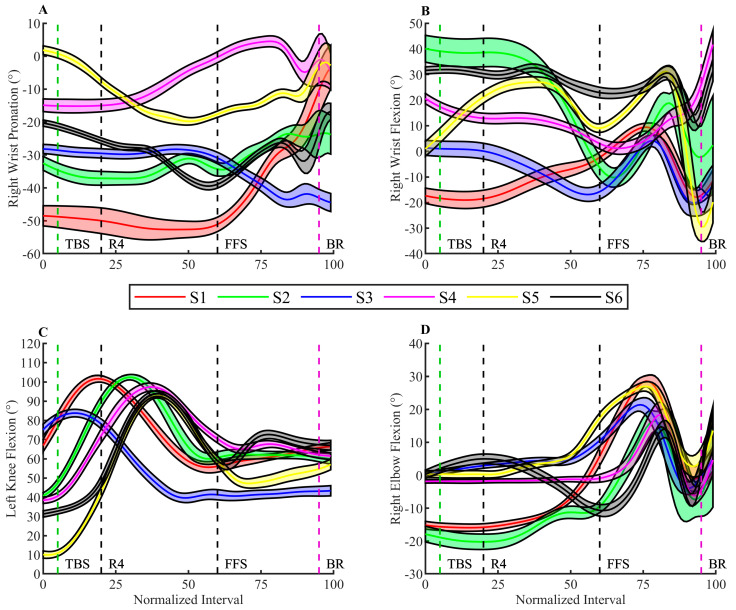
Shows joint angles mean ± SD for all participants individually without accounting for bouts. Black vertical lines are mean heel contact of right (R4) as the fourth step and FFS as the left and last step of the bowling delivery. Green and pink lines represent TBS and BR, respectively. (**A**) Right wrist pronation. (**B**) Right wrist flexion. (**C**) Left knee flexion. (**D**) Right elbow flexion.

For the movement pattern no differences were found before TBS. However, results showed changes in the dominant right arm for the bowlers in the last part of the bowling delivery (Figure 3). The right wrist and elbow motion was significantly more flexed in later bouts prior to BR (*p* < 0.001 and *p* = 0.004), respectively. Additionally, bowlers significantly increased the amount of pronation in the right wrist in later bouts at BR (*p* = 0.034). Furthermore, bowlers utilised the wrist range of motion differently from TBS to BR, with different wrist flexion and pronation prior to BR (cf. Figure 4). Nevertheless, bowlers remained consistent in their motion during the few milliseconds before the actual BR where the wrist range of motion was enabled similarly.

## 4. Discussion

The current study applied a novel field test approach to increase the biomechanical body of knowledge in elite ten-pin bowling. Here, movement characteristics, vertical GRF, and bowling parameters from bowling deliveries were obtained. The main findings were that elite bowlers lowered the BR_vel_, increased right wrist and elbow flexion prior to BR, and performed more pronation of the right wrist during BR, all in later bouts. Secondary findings were the borderline significant positive correlation between F_peakL_ and BR_imp_ and negative correlation between F_peakR_ and BR_vel_.

None of the hypotheses were confirmed in the present study. However, it is essential to recognise that the dynamic and evolving nature of sports techniques may have changed overtime due to advancements in training methodologies, equipment, and biomechanical understanding. Given the relatively scarce published work related to ten-pin bowling, it is conceivable that these changes may not have been observed scientifically but may be an unconscious knowledge for the professional athletes and their coaching staff. This in turn, may have produced conflicting results with the current literature which primarily dates back several decades. In particular, the use of in-field wearable IMU sensors for measuring sports performance have advanced various sports disciplines rapidly with new insights [20]. Nevertheless, a moderate positive correlation between BR_vel_ and B_score_ was seen, although this was not significant. Thus, the results presented by Razman et al. [8] could not be replicated. A lack of replication is probably due to the relatively small existing population of male elite bowlers in Denmark (*n* = 6). Additionally, the casual pin-fall measurement is limited in explaining the real bowling score compared to an actual game. The second hypothesis was the existence of a negative correlation between BR_imp_ and BR_vel_. The results revealed a small negative relationship as previously reported [8], but this was not significant. Previously in cricket, a positive correlation between horizontal breaking impulse and BR_vel_ has been reported [12]. However, the results from cricket were comparable to neither the present correlation between vertical BR_imp_ and BR_vel_ nor the peak deceleration of the foot during FFS and BR_vel_ [8]. It seems unclear if the same tendency seen in cricket [12] would be obtained if the horizontal BR_imp_ was measured in the present study. 

F_peakR_ foot was highly negatively correlated with the BR_vel_, which is an interesting observation. Such a relationship might be indicative of an increased vertical trajectory of the centre of mass, resulting in a less horizontal motion, giving a shorter temporal execution of the FFS. This finding is in line with previous observations where a shorter execution of the FFS resulted in higher BR_vel_. In addition, when comparing peak GRF in ten-pin bowling to that of baseball, peak GRFs seems to be decisive for BR_vel_, especially in the stride leg during the arm-cocking phase [21,22,23], whereas GRF seems to influence the BR_vel_ negatively in ten-pin bowling. Furthermore, according to our third hypothesis, the COM_vel_ in ten-pin bowling did not seem to have the same effect on the BR_vel_ as observed in cricket, where the initial velocity, acceleration, and deceleration seem much more crucial for BR_vel_ [10,11]. The inability to explain present observations, in accordance with the hypothesis, could suggest that the key to understanding the complexity of ten-pin bowling and obtaining advantages in performance strategies are positioned elsewhere. 

In the present study, it was observed that the BR_vel_ decreased over bouts as the test session progressed. However, no changes in peak GRF in the feet, COM_vel_, and BR_imp_ were observed. But several significant differences were observed in joint angles over bouts. Here, increased pronation of the dominant wrist was found, suggesting a greater utilization of the wrist’s range of motion throughout bouts. Supplementary, increased flexion of the right wrist and elbow was observed during the pendulum swing phase, which results in having the bowling ball tucked closer to the body. These behavioural changes might be performed as preparation to gain a better grip of the bowling ball, prior to the increased pronation during BR. It is unlikely that the changed kinematics over time are due to physiological fatigue [3,4], but rather a result of a conscious modification due to changing circumstances.

In ten-pin bowling the first 12 m of the 18.3 m long bowling lane is covered in an oil pattern. In the present study, the participants performed 72 bowling deliveries. Each delivery changes the dynamic of the oil pattern on the lane. The bowling ball absorbs oil as well as drags oil while moving, resulting in different traction between the bowling ball and lane at various locations as the test session prolongs. Assuming that the bowling athlete exerts his optimal power in each throw, then the total power applied is preserved. The total energy of the bowling ball is comprised of the linear kinetic energy and angular kinetic energy generated from the motion prior to BR. The linear kinetic energy in the moment of BR was found to be decreasing over bouts, as the BR_vel_ was reduced but the mass remained constant. Moreover, the angular kinetic energy most likely increases as more wrist pronation was observed, thereby, increasing the angular velocity in the wrist. Even though the rounds per minute of the bowling ball were not measured, it is reasonable to assume that additional wrist pronation resulted in more rotational energy in the bowling ball. More rotational energy allowed the bowling ball to rotate more and, in this way, the bowler compensated for the dynamic changes of the oil patterns as the test session progressed. To overcome the issue with changing dynamics of the oil pattern, three different methods may be proposed to the elite ten-pin bowler from the present results. Firstly, the bowler could maintain BR_vel_ to maintain the highest probability of a strike outcome. However, this method requires that the bowler manages to add more total rotational energy to the system as the bowling match prolongs. Secondly, the bowler could decrease the BR_vel_ gradually and maintain the same rotational energy to overcome the issue. In this solution, the bowler removes total energy in the form of linear kinetic energy as the bowling match prolongs. Lastly, the bowler could combine the first and second solutions. Here, the BR_vel_ and rotational energy gradually decrease and increase, respectively. The last solution was observed in the present study from the elite ten-pin bowlers.

If the bowling athlete exerts his optimal power, as assumed, the observed results and implication of these could be beneficial for ten-pin bowling athletes and coaches. Considering the dependency of the average bowling score on BR_vel_, the first proposed method would be the most intuitive solution to maintain the highest probability of strike outcome as higher velocity generates greater pin action and pin carry. Therefore, the skill to increase the system’s total rotational energy would be advantageous as the bowling match prolongs to compensate for the change in traction from repeated bowling deliveries. Ultimately, ten-pin bowlers should increase the total linear kinetic energy in general. Thus, as the match prolongs, a gradual increase in rotational energy is needed to maintain the highest probability of strike deliveries. Previously, it was shown that elite ten-pin bowlers outperformed semi-elite and non-elite bowlers with greater arm flexion as well as greater internal wrist rotation [24]. However, until now, no clear answer to why elite bowlers outperformed in exactly these parameters has been provided. Taking the changed kinematics over time, changed circumstances in traction from repeated bowling deliveries, and the assumption of optimal power into the equation, these observed changes must be performed as a solution to maintain the highest probability of strike deliveries. Therefore, it is recommended that elite ten-pin bowlers implement wrist and forearm training protocols that previously showed significant improvements on pronation and reduced motor control errors [25,26] to improve the system’s total energy. This proactive approach may not only enhance the competitive skill of the bowler but is also important for injury prevention. Ten-pin bowlers frequently suffer from De Quervain’s tenosynovitis along with arthritis symptoms in the wrist and finger joints [27]. Therefore, targeted training can mitigate injury risk and contributes to a competitive edge and overall well-being. Moreover, assessing muscle strength in players to discern and understand the potential impact on their preferred technique (i.e., Stroker, Cranker, or Tweener style bowling) may be beneficial for custom-made training schedules for different style bowlers, as demonstrated in various sports disciplines [28]. Additionally, when combined with a comprehensive kinematic analysis of different bowling styles, this approach can provide valuable insights for exactly what type of training is necessary, both dependent and independent on bowling style. 

In the present study, all participants exhibited a Tweener style of bowling. Tweener bowlers embody a blend of characteristics from both Crankers and Strokers. This amalgamation suggests potential intra-individual variations in biomechanical delivery patterns since Tweener bowlers navigate in the spectrum between power-focused Crankers and precision-oriented Strokers. In particular, utilisation of the bowlers’ wrist range of motion in the preparation window from TBS until BR was found to be different among the elite population in the current study (cf. Table 2 and Figure 4). These inherent differences among Tweener bowlers could influence the statistical significance of certain findings. Thus, acknowledging and delving into intra-individual variations could enhance the understanding of the diverse biomechanical profiles that may exist within the Tweener category. Therefore, further research is encouraged to explore the variations and unique characteristics among different bowling styles with a larger and more diverse sample size.

In the present study, bowlers were not allowed to switch ball, but such restrictions are not accurate for the true game. The effect of changing the ball on the kinematic execution during the test session is unclear. However, the changing circumstances in traction from repeated bowling deliveries would remain. Therefore, bowling coaches and athletes could experiment with both training protocols, as recommended, and change the bowling ball as a combined topic to increase the total energy to achieve advantageous strategies for optimal performance in ten-pin bowling. 

## 5. Conclusions

In summary, this study showed that elite ten-pin bowlers changed kinematics over time, with increased flexion in the dominant wrist and elbow prior to BR and increased wrist pronation during BR of the dominant wrist. These changes were most likely performed to compensate for the change in traction between the ball and the lane from repeated bowling deliveries. Bowlers maintained their total energy but increased the angular kinetic energy while decreasing the linear kinetic energy. This, in turn, caused the observed decrease in BR_vel_ as the test session prolonged. Ten-pin bowling coaches and athletes are recommended to experiment with wrist and forearm training protocols together with changing bowling ball dynamics to achieve advantageous strategies for optimal performance. The focus should be on increasing the total energy for both linear and angular kinetic energy.

## Figures and Tables

**Table 1:** Descriptive data of the measured bowling parameters over all six bouts. * Significant main effect over bouts, (*p* < 0.05). ^†^ Indicates a significant difference between bout 6. ^‡^ Indicates a significant difference between bout 5.

Variables	Bout 1	Bout 2	Bout 3	Bout 4	Bout 5	Bout 6
F_peakL_ (%BW)	166 ± 31	166 ± 31	164 ± 30	164 ± 29	166 ± 30	166 ± 30
F_peakR_ (%BW)	140 ± 18	140 ± 19	141 ± 18	139 ± 18	140 ± 19	141 ± 18
BR_imp_ (%BW⋅s)	77 ± 31	79 ± 29	80 ± 29	84 ± 28	79 ± 28	79 ± 30
COM_vel_ (m/s)	2.63 ± 0.35	2.63 ± 0.35	2.66 ± 0.29	2.67 ± 0.22	2.65 ± 0.30	2.59 ± 0.34
B_score_ (pin-fall)	8.5 ± 0.65	8.82 ± 0.47	8.93 ± 0.47	8.87 ± 0.49	9.32 ± 0.31	9.00 ± 0.38
* BR_vel_ (m/s)	8.37 ± 0.28 ^†,‡^	8.31 ± 0.33 ^†^	8.23 ± 0.36 ^†^	8.14 ± 0.35	8.09 ± 0.34	8.07 ± 0.38

**Table 2:** Descriptive data of intra-individual differences for the measured bowling parameters in the first bout and mean of the entire bout.

Variables	S1	S2	S3	S4	S5	S6	Mean ± SD
F_peakL_ (%BW)	173 ± 7	126 ± 3	170 ± 6	169 ± 3	139 ± 4	221 ± 7	166 ± 31
F_peakR_ (%BW)	147 ± 10	104 ± 3	147 ± 3	134 ± 8	154 ± 4	154 ± 3	140 ± 18
BR_imp_ (%BW s)	58 ± 18	75 ± 3	78 ± 6	78 ± 15	41 ± 9	134 ± 6	77 ± 31
COM_vel_ (m/s)	2.05 ± 0.30	2.68 ± 0.12	2.82 ± 0.04	2.37 ± 0.31	2.91 ± 0.05	2.96 ± 0.12	2.63 ± 0.35
B_score_ (pin-fall)	9.08 ± 0.95	8.83 ± 1.40	8.75 ± 1.42	8.17 ± 1.52	7.33 ± 2.69	8.83 ± 1.14	8.5 ± 0.65
BR_vel_ (m/s)	8.25 ± 0.07	8.52 ± 0.12	8.53 ± 0.10	8.76 ± 0.14	8.00 ± 0.14	8.16 ± 0.06	8.37 ± 0.28

**Table 3:** PPM correlation analysis used to assess relationship between dependent variables. The correlations coefficient and the corresponding *p*-values are presented. ^T^ Indicates a borderline significant trend (*p* = 0.057).

	F_peakL_ (%BW)	F_peakR_ (%BW)	BR_imp_ (%BW s)	COM_vel_ (m/s)	B_score_ (Pin-Fall)	BR_vel_ (m/s)
F_peakL_ (%BW)	1	0.62	0.80 ^T^	−0.05	0.25	−0.45
F_peakR_ (%BW)		1	0.11	0.04	−0.03	−0.80 ^T^
BR_imp_ (%BW⋅s)			1	0.19	0.09	−0.21
COM_vel_ (m/s)				1	−0.35	−0.45
B_score_ (pin-fall)					1	0.32
BR_vel_ (m/s)						1

## Data Availability

Not applicable.

## Abbreviations

The following abbreviations are used in the manuscript:

BRvel	Bowling ball release velocity
BR	Bowling ball release
TBS	Top of the backswing (highest bowling ball position)
FFS	Front foot slide (bowler slides, breaks before a foul line and releases the bowling ball)
COMvel	Centre of mass velocity
BRimp	Vertical breaking impulses (From start of FFS until BR)
IMU	Inertial measurement units
GRF	Ground reaction force
SD	Standard deviation
Bscore	Bowling score (measured as pins (0–10))
FpeakL	Peak GRF generated doing the FFS
FpeakR	Peak GRF generated doing the last heel contact before FFS
SPM1D	One dimensional statistical parametric mapping
